# Experimental and Numerical Study on the Penetration Performance of a Shaped Charge

**DOI:** 10.3390/ma15113899

**Published:** 2022-05-30

**Authors:** Yanan Du, Guanglin He, Weizhe Li, Kaipeng Wang

**Affiliations:** Science and Technology on Electromechanical Dynamic Control Laboratory, Beijing Institute of Technology, Beijing 100081, China; 3120205105@bit.edu.cn (Y.D.); 3120210177@bit.edu.cn (W.L.); 3120215105@bit.edu.cn (K.W.)

**Keywords:** shaped charge jet, penetration depth, numerical simulation, test verification

## Abstract

In guided ammunition, because a shaped energy jet warhead is located behind the control cabin (including the guidance cabin, the steering gear cabin, and the flight control cabin), the penetration order of a shaped energy jet is the control cabin and the target plate. In order to obtain maximum penetration depth by a shaped energy jet into a Q235 steel plate, the penetration performance of shaped energy jets was studied by numerical simulation and experimental verification. Firstly, the penetration performance of a warhead under different conditions at a certain explosion height is studied, which is the penetration performance of a Q235 steel plate with and without the control cabin. Secondly, the numerical simulation results are verified by experimental method. The numerical simulation and experimental results showed that, after penetration of the shaped energy jet warhead into the control cabin, it continued to penetrate the 20 mm-thick Q235 steel plate. At a certain explosion height, the maximum penetration depth of the shaped energy jet warhead into the Q235 steel plate was about 80 mm. Alongside the numerical simulation and experiment, the armor-breaking process of the shaped charge jet was analyzed theoretically. The results show that when the shaped energy jet warhead is located behind the control cabin, although the control cabin will have a certain impact on the penetration ability of shaped energy jet, the penetration performance of the residual jet still has the ability to penetrate light armor.

## 1. Introduction

A shaped charge, also known as a cavity charge, is a type of charge [1] with a concave charge cover at one end. After the charge explodes, the generated detonation wave is rapidly transmitted to the top of the charge cover along the axis and to the bottom of the charge cover through the force of high-temperature, high-pressure gas. At the same time, the charge cover is squeezed to the axis under the combined action of high-temperature, high-pressure gas, and the overpressure of the detonation products. The liner is extruded and deformed, colliding at the axis and forming a high-speed shaped jet [2]. The shaped charge jet has excellent armor-breaking performance and is widely used as anti-armor target ammunition [3] and in oil exploitation [4]. In terms of the penetration of a shaped charge jet into a target plate, Fan et al. [5] and others studied the penetration effects of double-layer shaped charge jets under the simulation conditions of Composition B (Comp-B) explosives with a density of 1.7 g/cm^3^ and a detonation velocity of 7980 m/s through a numerical simulation method. The simulation results showed that the C-J (Chapman–Jouguet) detonation velocity and the jet tip velocity both had an important impact on the DLSC’s penetration of the target. With an increase in the jet tip velocity, the penetration time first decreased and then increased. Chick and Hatt [6], Ding et al. [7], and Dou et al. [8], through numerical simulation and experimental methods, and taking a Comp-B explosive (with a density of 1.7 g/cm^3^ and a detonation velocity of 7980 m/s) as the internal charge, analyzed the penetration process of shaped charge jets into granite, ordinary strength concrete, and high strength concrete, and the linear relationship between the perforation diameter and the strength of the penetration target was obtained. Zhang et al. [9], through numerical research, and taking the cast TNT (2, 4, 6-trinitrotoluene) (with a low detonation velocity and a density of 1.63 g/cm^3^) as the internal charge, analyzed by a new angle model the formation of a shaped charge jet penetrating the target plate. Wang et al. [10] studied the jet formation and target penetration of a double-layered shaped charge (with TNT/JO-8 as the internal charge) through numerical simulation and experimental verification. The research results showed that the penetration ability of a double-layered shaped charge increases with an increase in the jet tip’s velocity and decreases with an increase in the Chapman–Jouguet (C-J) detonation velocity. Increasing the detonation velocity ratio, as well as the radial charge rate of the outer and inner charge, is conducive to promoting the formation of a detonation wave and improving the jet tip’s velocity.

In order to further improve the armor-breaking performance of shaped charge jets, scholars have committed to studying the theoretical models of jets when the target interacts with the shaped charge jet. In terms of the theoretical analysis of shaped charge jets, in reference [11], using PETN with a density of 523 cm^3^/10 g and a detonation velocity of 8083 m/s as the internal charge, Mickovic et al. studied the phenomena of an interaction between explosive reactive armor and a shaped charge jet [12,13,14], and produced a theoretical physical model of the interaction. Through numerical simulation [15,16] and experimental verification, it was concluded that, when the angle of attack is zero, the interaction between the jet and high detonation products reduces the penetration depth into the target plate. Elshenawy et al. [17] proposed a numerical method to determine the exact position of the caliber shaped charge jet (VO). Through the method of numerical simulation and experimental verification, it was concluded that the maximum difference between the penetration depth and the penetration depth calculated by this method is less than 4%. In the test, RDX-C4, TNT, PETN, and HMX internal charges were used, and a PHYWE hydraulic press was used to suppress the explosive under a high specific pressure of 20 kN/cm^2^, so as to draw the test conclusion. Chen et al. [18] simulated a shaped energy jet by using the improved CE/SE scheme, combined with a high-resolution interface tracking method and a fluid boundary treatment. In the test, TNT, and Comp-B explosives with detonation velocities of 6930 m/s and 7980 m/s, respectively, were used as internal charges. However, they did not compare the calculated results on, for example, jet flow energy and velocity gradient, with relevant experiments. Wojewodka et al. [19] used LS DYNA software to simulate the jet formation process of linear shaped charges. In the numerical simulation, RDX (hexahydro-1, 3, 5-trinitro-1, 3, 5-triazine) was used as the main charge, and the detonation velocity was set to 8490 m/s. Liu et al. [20] and Wu et al. [21] both studied a theoretical method of calculating the interaction between the shaped energy jet and the target plate during penetration of the target plate through theoretical analysis and a jet penetration test. In the test, 8701 explosives and Comp-B explosives, with detonation velocities of 8390 m/s and 7980 m/s, respectively, were used as internal charges. The results showed that both the thickness of the target plate and the distance between the target plates will have a certain impact on the performance of the shaped energy jet penetrating the target plate. Wang et al. [22] studied the jet formation process and the penetration performance of different material liners through a combination of numerical analysis and experiments. The two materials used were copper and 1045 steel. In the jet test, a Comp-B explosive (with a density of 1.7 g/cm^3^ and a detonation velocity of 7980 m/s) was used as the internal charge. The research results showed that the diameter of the jet tip produced by the two materials was similar. However, the change gradient of the steel jet’s diameter was larger than that of the copper jet. At the same time, copper has great ductility under tension because of its good tensile properties. Greater ductility leads to longer jets, which can improve their penetration ability. Metals with good plasticity, such as pig iron, aluminum, tungsten [23] and red copper [24], are used as liner materials. By means of numerical simulation and experimental verification, Zochowski et al. [25] studied the factors affecting the formation process of a shaped charge jet and underestimated or overestimated its penetration ability. In the test, an A-IX-1 explosive was used as the main charge. The results showed that improvements in the material properties of warhead components can greatly improve the penetration reliability of the warhead. Liu et al. [26] studied the reaction degree of Comp-B explosives (with a density of 1.7 g/cm^3^ and a detonation velocity of 7980 m/s) through numerical simulation and experimental verification. The research results showed that the reaction degree of an explosive can be analyzed from the fragment velocity, fragment puncture pattern, and damage characterization of the bottom target. Michael L. Hobbs [27] and other researchers conducted complex thermal decomposition modeling and analysis on the molten explosive Comp-B (composed of RDX and TNT) through numerical simulation and experimental verification [28,29,30], determining the reactivity changes caused by melting and dissolution.

It can be seen from the above references that researchers have conducted extensive research on the penetration performance and theoretical models of shaped energy jets. The penetration effect of a shaped energy jet on different dielectric targets has been studied in the literature [5,6,7,8,9], as has the formation and simulation of shaped energy jets [11,12,13,14,15,16,17,18,19]. According to the conclusions of one study [18], a simulation of a shaped charge jet can be used to analyze the jet formation process to a certain extent, and the jet formation mechanism can be used to help improve the penetration efficiency of a shaped charge jet warhead. However, because this is not combined with the test results, the research results are uncertain. Therefore, it is necessary to study the penetration performance and jet behavior of shaped charge jet warheads more accurately. Firstly, the penetration performance of the post-shock shaped charge jet warhead (after penetrating the control cabin), as well as the formation and fracture processes of the jet, were studied by numerical simulation. Secondly, through the combination of numerical simulation and experimental verification, the penetration depths of the target plates, with and without the control cabin, respectively, were studied, and the jet behavior during the process of penetration was analyzed in detail.

## 2. Structural Design

### 2.1. Warhead Structure Design

**Figure 1:**
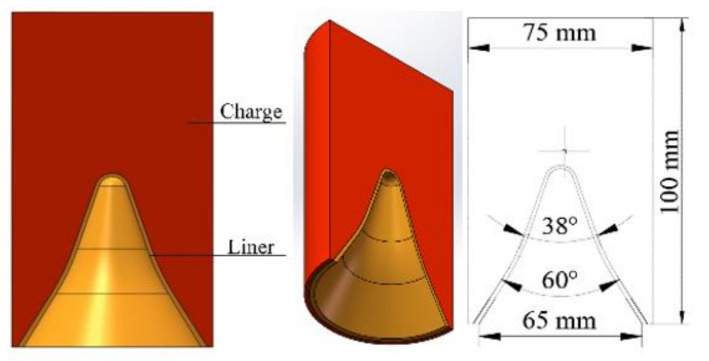
Structure of the shaped charge warhead.

The charge diameter of the shaped charge armor-piercing warhead designed in this study was 75 mm and the charge height was 100 mm. In order to improve the jet length and penetration depth as much as possible, the liner structure was designed with a variable wall thickness and cone angle. That is, the thickness of the liner gradually increased from the top to the bottom of the liner, in which the thickness at the cone angle at the top of the liner was 1.4 mm and the wall thickness at the bottom of the liner was 2 mm; the angle of the shaped charge liner gradually increased from the top to the bottom. The cone angle from the top to the middle of the shaped charge liner was 38° and the cone angle from the middle to the bottom of the shaped charge liner was 60°. The height of the liner was 68 mm. The design of the shaped charge warhead is shown in Figure 1.

### 2.2. Target Equivalent Design

A shaped charge jet warhead is mainly used to attack the top armor or side armor of armored vehicles. The thickness of the top armor and side armor of armored vehicles, infantry combat vehicles, command and control vehicles, ammunition vehicles, and other vehicles is generally 12~16 mm. Multiplied by the equivalent coefficient 1.2, the equivalent thickness is 19.2 mm. Therefore, in the numerical simulation, the target plate was 20 mm thick; a Q235B steel plate 100 mm thick × 100 mm was simulated.

### 2.3. Equivalent Design of Control Cabin

The equivalent design of the simulated missile’s guidance and control cabin included the following: two Q235B steel plates (3 mm thick and 100 mm thick), which were placed on the uppermost layer of the test chamber; a 100 mm Q235B steel plate (analogous to the steering gear and optical lens medium); two pieces of nylon 66 plastic board (2 mm thick, 100 mm × 100 mm; analogous to the circuit board); one aluminum plate (10 mm thick, 100 mm × 100 mm; simulating the thermal battery, the missile-borne computer, and the aluminum structural components of the flight control cabin). This was used to simulate the cabin section in front of the warhead. Each simulated medium was stacked together, with a total height of 20 mm.

**Figure 2:**
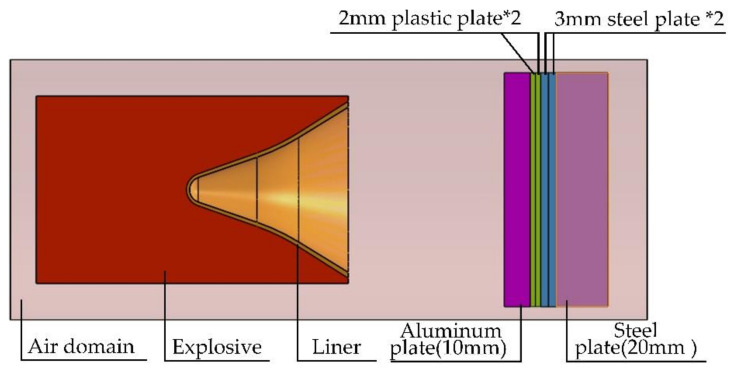
Structural model of penetration into the control cabin and target plate.

**Figure 3:**
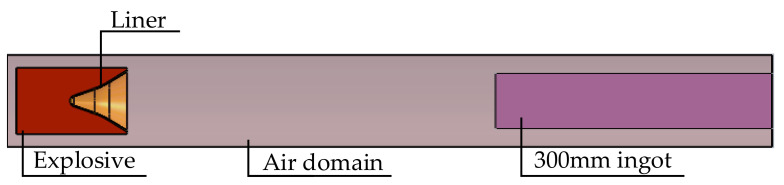
Structural model of penetration into a steel ingot.

A structural diagram of the shaped energy jet warhead penetrating into the control cabin and the Q235B steel target is shown in Figure 2, and a structural diagram of the shaped energy jet warhead penetrating into a Q235B steel ingot is shown in Figure 3.

## 3. Numerical Simulation Model

The numerical simulation model consisted of the explosive, the liner, air, the simulated control cabin, and the Q235 steel target. The charge, liner, air domain, aluminum plate, and Q235 steel target were uniformly continuous media. The calculation used top center initiation, and the charge’s structure was axisymmetric. In the numerical simulation, the multi-material arbitrary Lagrange Eulerian (ALE) algorithm and the fluid structure coupling method were used to realize the numerical simulation in LS-DYNA.

### 3.1. Numerical Algorithm

In the numerical simulation model, the explosive, liner, and air are fluid structures, which are modeled by a Euler grid. This element adopted a multi-material group ALE algorithm to simulate the cabin and Q235 steel target as solid structures, modeled by Lagrange grids. The interaction between the fluid structure and solid structure materials was modeled by a fluid structure coupling algorithm.

The ALE algorithm can accurately simulate the characteristics of a high strain rate, high pressure, and nonlinear deformation, and can avoid serious mesh distortion. The charge, liner air, aluminum plate, and Q235 steel plate were calculated with the multi-material group ALE algorithm and processed by the Lagrange algorithm. This method effectively solves the interaction between a fluid structure and a solid structure by coupling compressible fluid with solid-structure materials.

### 3.2. Finite Element Model and Parameters

The three-dimensional structure model of the warhead was imported into HyperMesh to mesh the liner, explosive, air domain, and target plate, with a grid size of 1 mm × 1 mm. The liner grid, explosive grid, and air domain grid were mainly hexahedral and prismatic pentahedral grids. As the ALE algorithm was adopted for the shaped charge jet, common nodes were required between the liner grid and the explosive grid, between the liner grid and the air domain grid, and between the charge grid and the air domain grid. The control cabin and target plate all adopted a hexahedral grid. Due to the small jet diameter, the penetration area was mainly concentrated in the center of the target plate. Therefore, in the radial direction of the target plate, the grid size gradually increased from the center to the edge, and the grid size remained unchanged in the axial direction of the target plate. Since the model had an axisymmetric structure and accounted for computation and performance, a 1/4 model was used for numerical simulation to reduce the computation time. Because the numerical simulation adopted the fluid structure coupling algorithm, there must be coincidence between the fluid grid and the solid grid. The initiation mode of the numerical model is central single-point initiation. The initial stress, initial temperature, and initial velocity of the warhead were all 0.

**Figure 4:**
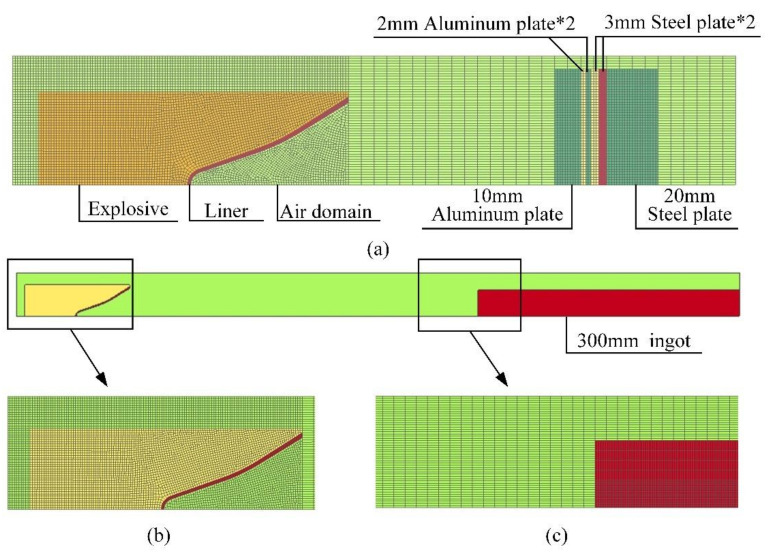
Finite element model. (**a**) Finite element model of the warhead penetrating the control cabin and target plate, with 177,536 grids; (**b**) finite element model of the warhead penetrating into a 300 mm Q235 ingot, with 337,420 grids; (**c**) Grid condition at the junction of ingot and air domain.

The finite element model adopted a symmetrical boundary, and some numerical models are circularly symmetrical. At the same time, there is no reflection boundary, sliding boundary, or failure criterion symmetry. Since the penetration model was an axisymmetric structure, in order to reduce the amount of calculations, a quarter-scale finite element model was established, as shown in Figure 4.

### 3.3. Material model and Parameters

The liner material involved in this study was red copper, and the material parameters of the liner are shown in Table 1. The liner was modeled by the Johnson–Cook material model, which is widely used in penetration research. The yield stress is defined as:

(1) $$\sigma_{y}=\left(A+B\varepsilon_{p}^{n}\right)\left(1+C\ln{\dot{\varepsilon }}^{*}\right)\left[1-{\left(T^{*}\right)}^{m}\right]$$

where *A*, *B*, *n*, *C*, and *m* represent the initial yield strength, the strain strengthening index, the strain rate sensitivity coefficient, the hardening index, and the temperature softening index, respectively. These material parameters are usually determined through experiments. Moreover, $\varepsilon_{p}$ is the effective plastic strain and ${\dot{\varepsilon }}^{*}$ is the effective plastic strain rate. For VP = 0, it is defined as ${\dot{\varepsilon }}^{*}=\dot{\bar{\varepsilon }}/EPSO$, the effective total strain rate normalized by quasi-static threshold rate; for VP = 1, it is defined as ${\dot{\varepsilon }}^{*}={\dot{\bar{\varepsilon }}}^{p}/EPSO$, the quasi-static normalized effective total strain rate threshold rate. The homologous temperature is defined as $T^{*}=\left(T-T_{r}\right)/\left(T_{m}-T_{r}\right)$, where t is the current temperature, and $T_{m}$ and $T_{r}$ are the melting temperature and room temperature, respectively.

In order to describe the state of the material at a high strain rate, the Gruneisen state equation was used. The Gruneisen state equation with cubic shock velocity and particle velocity defines the pressure of compressed materials as:

(2) $$p=\left\{\begin{array}{c} \frac{\rho_{0}C^{2}\mu \left[1\;+\;\left(1\;-\;\frac{\gamma_{0}}{2}\right)\mu \;-\;\frac{a}{2}\mu^{2}\right]}{{\left[1\;-\;\left(S_{1}\;-\;1\right)\mu \;-\;S_{2}\frac{\mu^{2}}{\mu \;+\;1}\;-\;S_{3}\frac{\mu^{3}}{{\left(\mu \;+\;1\right)}^{2}}\right]}^{2}}+\left(\gamma_{0}+a\mu \right)E\left(\mu \geq 0\right)\\ \rho_{0}C^{2}\mu +\left(\gamma_{0}+a\mu \right)E\;\;\;\;\left(\mu \leq 0\right) \end{array}\right.$$

where *C* is the intercept of the $v_{s}-v_{p}$ curve; $S_{1},\;S_{2}$, and $S_{3}$ are the coefficients of the slope of the $v_{s}-v_{p}$ curve, and $v_{s}$ and $v_{p}$ are the shock velocity and particle velocity, respectively; $\gamma_{0}$ is the Gruneisen gamma; a is the first-order volume correction to $\gamma_{0}$; $\mu =\rho /\rho_{0}-1$ and E represents the internal energy.

The PLASTIC_KINEMATIC model was adopted for the materials of the top armor, the side armor steel plate, and the guidance control cabin of the simulated armored vehicle. Its material parameters are shown in Table 2.

The explosive studied in this work was Comp-B [18]. The high-energy material model adopted was the JWL state equation, and its material parameters are shown in Table 3. The pressure is expressed as:

(3) $$p_{E}=A\left(1-\frac{\omega }{R_{1}V}\right)exp\left(-R_{1}V\right)+B\left(1-\frac{\omega }{R_{2}V}\right)exp\left(-R_{2}V\right)+\frac{\omega E_{0}}{V}$$

where $p_{E}$ is the detonation pressure, $V=1/\rho_{0}$ is the relative volume, $\rho_{0}$ is the density of the explosive, $E_{0}$ is the specific internal energy per unit of mass, and *A*, *B*, $R_{1},\;R_{2},$ and ω are the material constants.

## 4. Numerical Simulation Results

**Figure 5:**
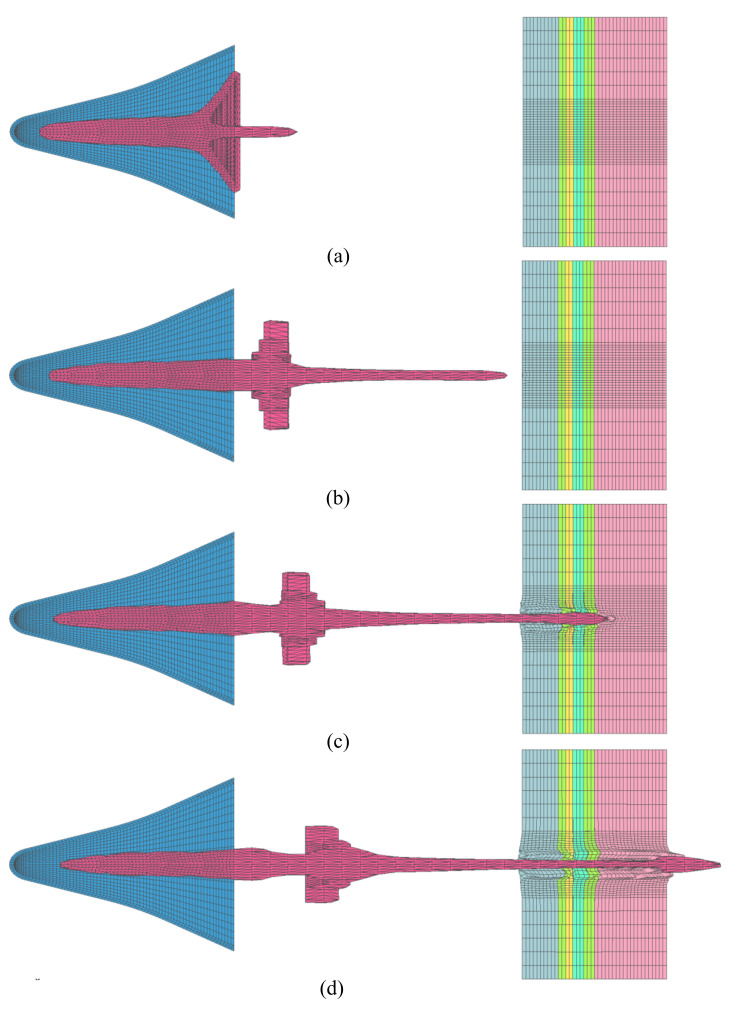
Penetration of the shaped charge jet into the simulated cabin target. (**a**) Jet condition when t = 20.5 μs; (**b**) Jet condition when t = 28.5 μs; (**c**) Jet condition when t = 33.5 μs; and (**d**) Jet condition when t = 39.5 μs.

The finite element model established above was generated into a k file and solved in the LS-DYNA solver. The penetration results when the shaped charge jet warhead was located behind the control cabin and the explosion height was twice the diameter of the liner are shown in Figure 5.

In Figure 5a, at t = 28.5 μs, the shaped jet had initially formed and showed a stretching trend. At t = 30.5 μs (Figure 5b), the front part of the jet formed at that time began to extend and elongate in the air. At t = 33.5 μs (Figure 5c), the jet penetrated the warhead control cabin and began to collide with the Q235 steel plate. After the collision, the jet had not consumed all the energy. Although the remaining energy could not break armor further, it could widen the aperture. This part of the jet expanded around and finally adhered to the hole wall under the impetus of subsequent jets. Therefore, the jet’s perforation of the target plate was larger than the jet diameter. At t = 38.9 μs (Figure 5d), the jet head penetrated the 20 mm-thick Q235 steel plate. At that time, the jet remained intact and there was no necking or fracture. Therefore, the jet still had penetration ability. The jet’s perforation diameter was 32 mm. The numerical simulation results showed that the shaped charge jet warhead could continue to penetrate the 20 mm-thick Q235 steel plate after penetrating the control cabin.

**Figure 6:**
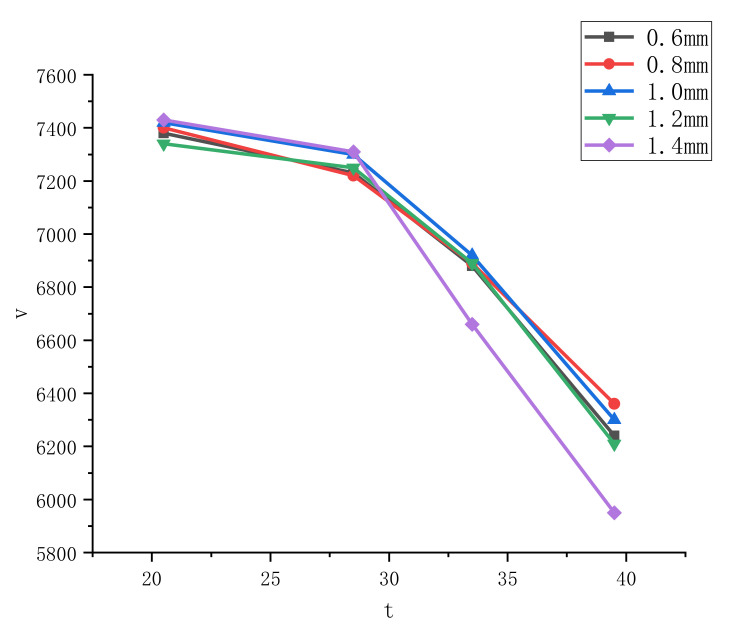
Jet velocity under different grid sizes.

In the numerical simulation, in order to verify the influence of the finite element mesh size on the numerical analysis results, five groups of meshes of different sizes were simulated for the same model. The mesh sizes of the five groups were 0.6 mm, 0.8 mm, 1.0 mm, 1.2 mm, and 1.4 mm. The simulation results are shown in Figure 6.

In Figure 6, at time t = 20.5 μs, the jet velocity for a grid size of 0.6 was 7380 m/s, 0.8 was 7400 m/s, 1.0 was 7420 m/s, 1.2 was 7340 m/s, and 1.4 was 7430 m/s. The maximum velocity was 7430 m/s, the minimum velocity was 7340 m/s, and the maximization rate of jet velocity was 1.2%. At time t = 28.5 μs, the jet velocity for a grid size of 0.6 was 7230 m/s, 0.8 was 7220 m/s, 1.0 was 7300 m/s, 1.2 was 7250 m/s, and 1.4 was 7310 m/s. The maximum velocity was 7310 m/s, the minimum velocity was 7220 m/s, and the maximum change rate of jet velocity was 1.2%. At time t = 33.5 μs, the jet velocity for a grid size of 0.6 was 6880 m/s, 0.8 was 6890 m/s, 1.0 was 6920 m/s, 1.2 was 6890 m/s, and 1.4 was 6660 m/s. The maximum velocity was 6920 m/s, the minimum velocity was 6660 m/s, and the maximum change rate of jet velocity was 3.7%. At time t = 39.5 μs, the jet velocity for a grid size of 0.6 was 6240 m/s, 0.8 was 6360 m/s, 1.0 was 6300 m/s, 1.2 was 6210 m/s, and 1.4 was 5950 m/s. The maximum velocity was 6360 m/s, the minimum velocity was 5950 m/s, and the maximum change rate of jet velocity was 6.4%. It can be observed that the change of jet velocity tended to be gentle under different grid sizes at the same time. Therefore, the change of grid size has little effect on jet velocity.

**Figure 7:**
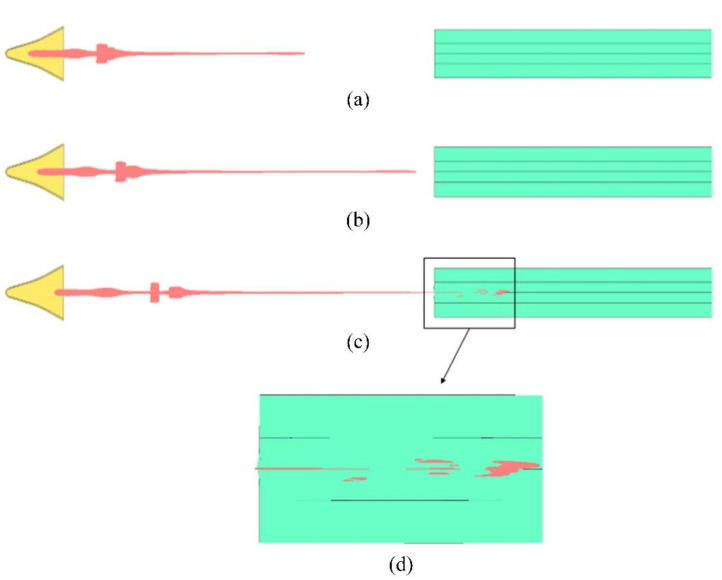
Numerical simulation results of the shaped charge jet directly penetrating the target. (**a**) Jet condition when t = 52.5 μs; (**b**) Jet condition when t = 62.5 μs; and (**c**) Jet condition when t = 77.5  μs; (**d**) Jet head condition when t = 77.5  μs.

It can be seen from the above that the grid size had little effect on jet velocity. Therefore, a 1 mm grid is still used when there is no simulation cabin. When there is no simulated cabin, the penetration results of the shaped charge jet, with a blast height of 6× the diameter of the liner directly penetrating a steel ingot, are shown in Figure 7.

Figure 7a shows t = 52.5 μs, when the jet had completely formed and extended in the air. At t = 62.5 μs (Figure 7b), the shaped energy jet extended to a certain extent in the air; it can be seen that the jet diameter was becoming smaller and smaller. At t = 77.5 μs (Figure 7c), the shaped energy jet penetrated the Q235 steel ingot. When the penetration depth reached 80 mm, the jet broke, and a small section of the broken jet continued to turn and deviate from the axis. Due to the high strength of the Q235 steel ingot without a layered target, the residual penetration energy of the broken shaped energy jet was insufficient, and the stress state gradually disappeared. The residual jet remained in the hole wall and expanded the hole’s diameter. The jet’s perforation diameter was 32 mm. Therefore, from the numerical simulation results, it can be seen that the effective depth of a shaped charge jet directly penetrating into a steel ingot is 80 mm under a fixed explosion height of 6×.

## 5. Test Verification

In order to study the penetration performance and jet behavior of a shaped charge jet warhead located behind the control cabin, two tests were carried out.

### 5.1. Penetration Simulation Cabin and Target Plate Test

#### 5.1.1. Test Setup

**Figure 8:**
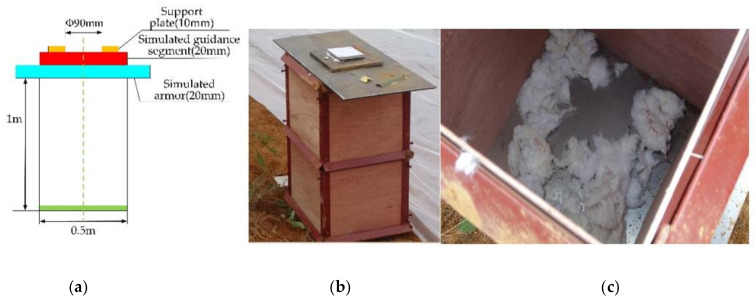
Layout of the test device. (**a**) The schematic diagram of the experimental device; (**b**) The test chamber; and (**c**) The cotton yarn in the test chamber.

A schematic diagram of the device used to test damage to the interior of the target (after the shaped charge jet warhead has broken the armor) is shown in Figure 8a, and the test setup is shown in Figure 8b. There is no limit between the explosive and the liner. The explosive is filled by filling. The total mass of the explosive is 709 g. The warhead was placed above the test chamber, and the distance between the warhead and the simulation control cabin section was equal to the charge diameter (i.e., one times the charge diameter). Cotton dipped in gasoline was placed at the bottom of the target frame, as shown in Figure 8c. During the test, the shaped charge was fixed above the target plate, aligned with the supporting steel plate (with Φ90 mm through the hole in the middle), and the shaped charge was kept perpendicular to the target plate.

#### 5.1.2. Test Result

**Figure 9:**
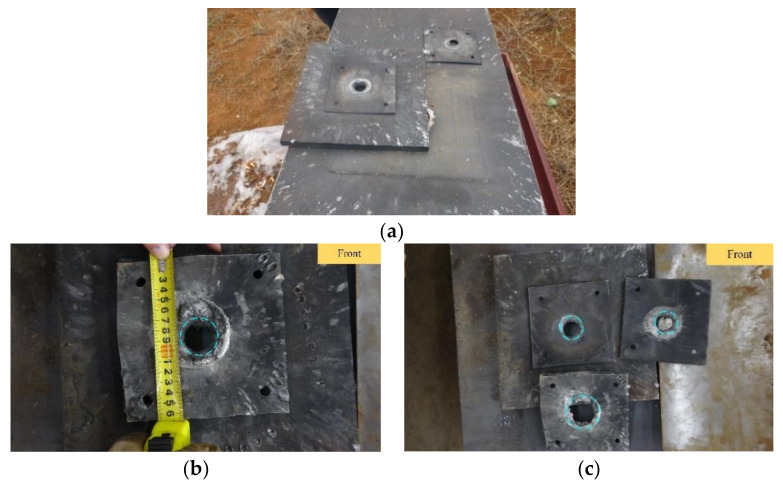
Penetration target test results. (**a**) Image of the recovered steel target; (**b**) measurement of the jet’s perforation size; and (**c**) the results of the steel target penetration test.

Once the test was ready, we detonated the warhead. After the test, we recovered the steel target and analyzed the penetration. Under the impact of the shaped energy jet, edge pits and subsequent through-holes were formed on the steel target. Their dimensions were measured in detail. The recovered steel target is shown in Figure 9.

It can be seen from the figure that after the middle of the five-layer equivalent simulation guidance and control cabin was penetrated by the warhead jet, the 20 mm-thick Q235 steel plate (simulating the top armor and side armor of an armored vehicle) was broken, and the jet’s perforation diameter was about 35 mm. Therefore, when the shaped jet warhead is located behind the control cabin—although the control cabin will have a certain impact on the penetration ability of the shaped jet—the penetration performance of the residual jet still has the ability to penetrate light armor.

### 5.2. M Maximum Penetration Depth Test of Shaped Charge Jet

#### 5.2.1. Test Setup

**Figure 10:**
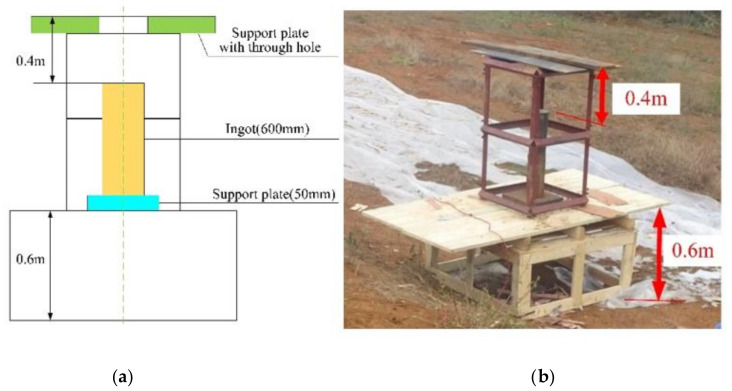
Layout of the test device. (**a**) The schematic diagram of the experimental device; (**b**) Layout of test target frame.

The test setup for direct penetration by the warhead into a steel ingot is shown in Figure 10; images of the warhead’s direct penetration into the steel ingot test device are shown in Figure 10a, and measurement of the penetration is shown in Figure 10b. By observing the after-effects of the shaped charge jet breaking armor, it was found that the fragments were mostly concentrated in the lower part of the target plate. In order to better collect the fragments, a wooden box and a wooden board were placed under the target frame, and the overall target frame was increased by 600 mm. A 20 mm-thick Q235B steel plate was placed in the target frame, a wooden plate was placed on the Q235 steel target with a through-hole in the middle, a 50 mm-thick steel plate was placed at the center of the bottom of the target frame, and a 600 mm-thick steel ingot was placed on the steel plate, aligned with the center of the steel plate’s through-hole on the target frame, marking the center position.

#### 5.2.2. Test Results

**Figure 11:**
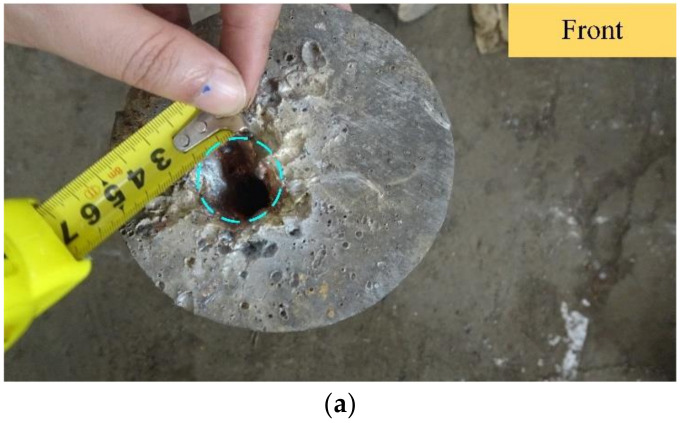

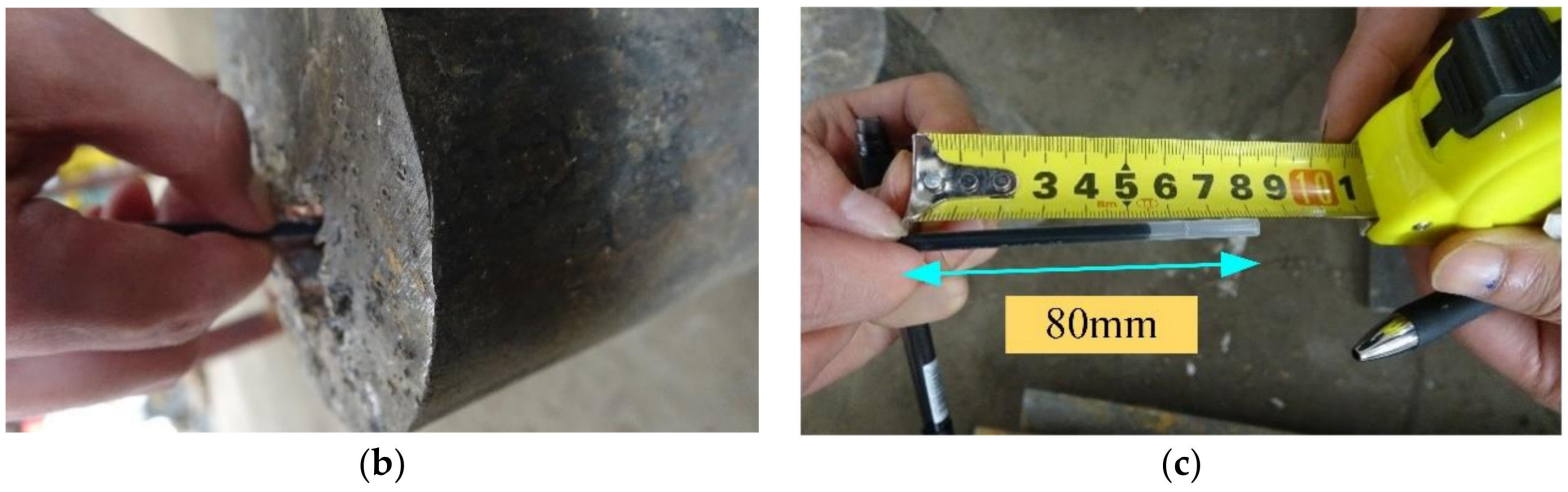
Ingot penetration results. (**a**) Measurement of the hole caused by the shaped energy jet; (**b**) measurement of the shaped energy jet’s penetration depth; and (**c**) depth of the shaped energy jet.

Once the test was ready, we detonated the warhead. We then recovered the steel target and measured the inlet diameter and depth of the shaped charge jet’s penetration into the steel ingot. The measurement results are shown in Figure 11.

The test results showed that the metal jet (formed by the warhead) formed a blind hole with a diameter of about 15 mm and a depth of about 80 mm in the ingot. It can be seen that when the shaped charge jet warhead directly penetrated the steel target at 6× the blast height, the maximum penetration depth was 80 mm.

## 6. Discussion

### 6.1. Phenomenological Description of Jet Formation and Penetration

**Figure 12:**
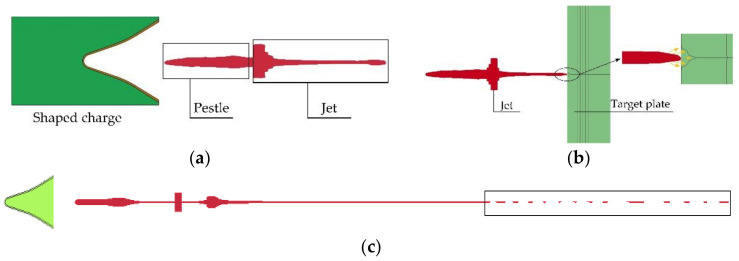
Jet penetration process. (**a**) The formation model of shaped charge jet; (**b**) The head motion model of shaped charge jet; and (**c**) The fracture model of shaped charge jet.

According to the numerical simulation results of the shaped energy jet penetrating the simulated cabin and steel target, and directly penetrating into the steel ingot, the penetration process of the shaped energy jet into the target plate can be divided into three stages, as shown in Figure 12a–c. Figure 12a shows the formation process of a shaped charge jet driven by the explosion. It can be seen from the figure that the liner moves successively to the axis of symmetry under the action of the explosive to form two parts: the jet and the pestle. Figure 12b shows the armor-breaking process of the shaped energy jet. When the shaped energy jet collides with the target plate, the collision velocity exceeds the velocity of sound in steel and copper. The jet’s diameter is very small, and the sparse wave is transmitted rapidly to create an opening. In order to make the jet’s shape clearer, Figure 12c shows only the jet, showing that the opening jet expands around under the impulse of the subsequent jet. When it penetrates to a certain depth, the jet necks, and breaks. After the jet overturns, it cannot break armor at all, and finally adheres to the hole wall.

### 6.2. Analysis of Jet Formation

**Figure 13:**
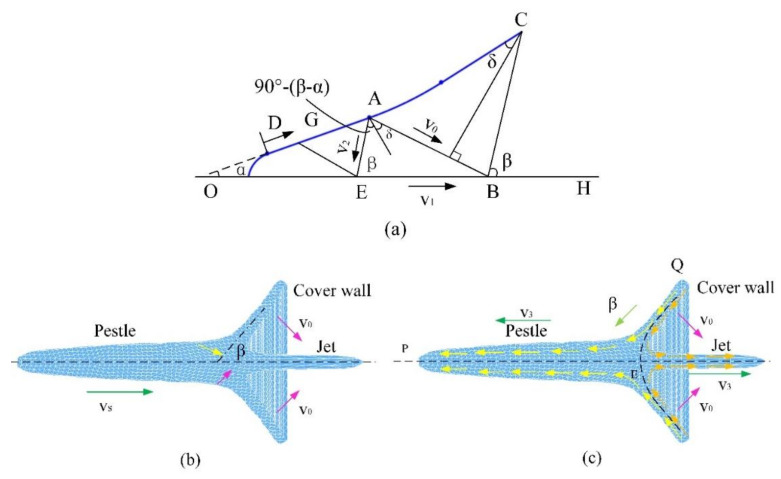
Schematic diagram of the jet formation. (**a**) Calculation graphics; (**b**) Static coordinate system; and (**c**) Moving coordinate system.

The initial pressure of the detonation wave, generated by the explosion reaching the wall of the liner, can reach hundreds of thousands of atmospheres, and the liner moves towards the axis. Through the numerical simulation and the test in this work, Figure 13a illustrates the calculation diagram of the shaped jet formation after the explosion, where OC is the initial position of the cover wall and α is the half cone angle. When the detonation wave reaches Point A, Point A begins to move with a velocity of $v_{0}$ (called the pressing speed), and the direction is proportional to the normal surface of the liner δ (called the deformation angle). When Point A reaches the axis, the detonation wave reaches Point C, Section AC moves to position BC, and the angle between BC and the axis is *β*. This is called the pressing angle. In the jet formation stage, the liner wall changes from CAE to CB, the collision point moves from point E to point B, and the moving speed is $v_{1}$. Assuming that the velocity *D* of the detonation wave sweeping through the wall cover is constant, a geometric relationship of steady motion can be obtained, as shown in Equations (4) and (5).

(4) β=α+2δ, 

(5) $$\sin\delta =\frac{v_{0}}{2D},\;$$

The jet formation diagram near the collision point is shown in Figure 13a; that is, in the static coordinate system, the wall cover moves to the axis at a speed of $v_{0}$. When it reaches the collision point, it is divided into the pestle and jet. The pestle moves at a speed of $v_{s}$, the jet moves at a speed of $v_{j}$, and the collision Point E moves at a speed of $v_{1}$. The jet’s motion at the collision point is shown in Figure 13b; that is, the jet’s motion observed at the angle of the collision point. It can be seen that the wall cover moves towards the collision point at a relative speed of $v_{2}$ and then divides into two strands: one to the left of the impact point and the other to the right of the impact point. The outer layer of the cover wall moves to the left of the collision point to become a pestle, and the inner layer of the cover wall moves to the right of the collision point to become a jet. According to the theory of hydrodynamics, the fluid motion of the shaped energy jet can be described by a Bernoulli equation; that is, the sum of pressure and kinetic energy density along the streamline is a constant. For Point Q on the cover wall and Point P of the pestle, Equation (6) can be obtained:

(6) $$p_{P}+\frac{1}{2}\rho v_{3}^{2}=p_{Q}+\frac{1}{2}\rho v_{2}^{2},\;$$

where $p_{P}$ and $p_{Q}$ are the static pressure at Point P and Point Q in the fluid, respectively, and ρ is the fluid density.

During jet formation, *δ* is a function of the cover element x for the shaped charge structure, *δ* (x). The expression is:

(7) $$\delta \left(x\right)=arc\sin\left[\frac{v_{0}}{2D}\cos\left(\alpha +arc\tan\frac{h-x\tan\alpha }{x+S}\right)\right],\;$$

Therefore,

(8) $$\tan\beta \left(x\right)=\tan\beta \left(v_{0},\;v_{0}^{\prime },\;x,\;\alpha ,\;D,\;S,\;h\right),\;$$

where, *α*, *S*, and *h* are determined by the charge structure, *D* is the detonation speed, and $v_{0}$ is the pressing speed.

**Figure 14:**
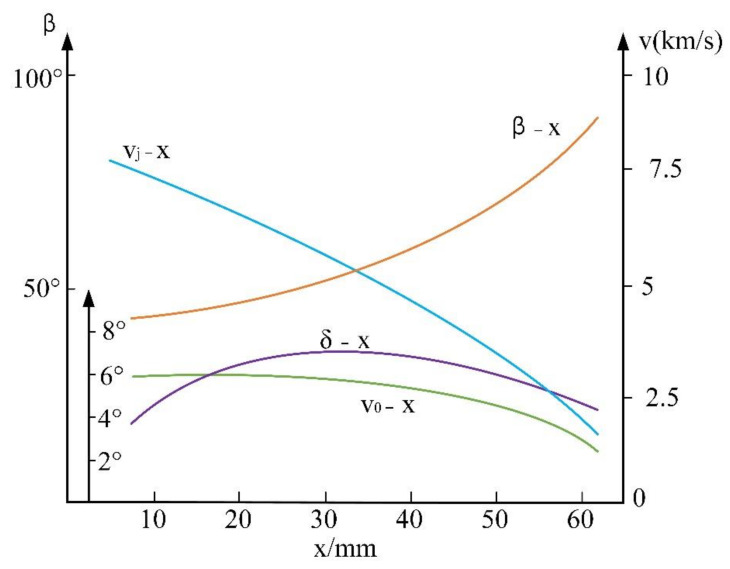
Distribution of the main parameters in the jet formation process.

Figure 14 shows the distribution of the main parameters in the jet formation process, where x is the distance between the microelement of the liner and the top, $v_{j}$ is the jet’s velocity, β is the pressing angle, δ is the angle between the deformation foot (i.e., the direction and the normal of the liner surface), and $v_{0}$ is the microelement’s pressing velocity. It can be seen from the curve that, when changing from the top of the liner to the bottom, the pressing speed $v_{0}$ of each microelement changes little and decreases faster. When it is close to the bottom of the cone, *δ*, the angle changes little. The change in the pressing angle is larger, and the rate of change along X axis is more uniform. Variation of several parameters shows the instability of the jet formation process.

### 6.3. Analysis of the Jet’s Armor-Breaking Process

The armor-breaking process of the jet is mainly divided into three stages [22,23,24]: the pit-opening stage, the quasi-steady stage, and the termination stage. The opening stage is the beginning of armor-breaking, where the jet head collides with the target plate, the shockwave is transmitted into the target plate and the jet, and three high areas (high temperature, high pressure, and high strain rate) are established in the target plate. After the pit-opening stage, the jet penetrates the target plate through the three high areas, and the collision pressure is small. In this stage, the energy distribution of the jet is slow, and there is little change in either the armor-breaking parameters or the diameter of the target hole (which is basically independent of the armor-breaking time)—this stage is called the quasi-steady stage. The termination stage is more complex: the jet’s velocity decreases, as does the armor-breaking velocity. At the same time, the jet necking and fracture phenomena appear in the later stage of armor-breaking, which also speeds up the arrival of the armor-breaking termination stage.

**Figure 15:**
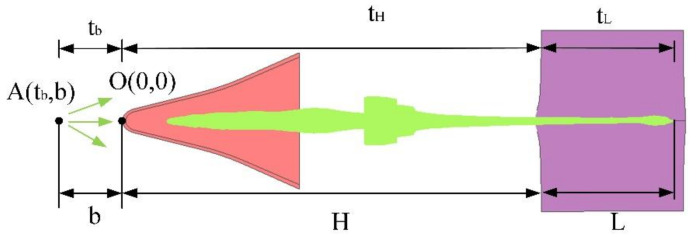
Simplified quasi-steady calculation model of armor-breaking by the jet.

According to the quasi-steady hydrodynamics theory, it is assumed that all the jet microelements start from Point A at the same time but have different initial velocities. With the passage of time, the jet continues to elongate due to the velocity difference, and the jet’s velocity is linearly distributed along the length. The numerical simulation model of the shaped charge jet penetrating the target plate was simplified to obtain a simplified quasi-steady calculation model of armor-breaking by a jet, as shown in Figure 15. The coordinates of Point A are $\left(t_{b},\;b\right)$ and the coordinates of O at the top of the liner are (0, 0).

According to the geometric relationship in the calculation model, we can get:

(9) $$\left(t_{H}+t_{L}-t_{b}\right)=L+H-b,\;$$

where *H* is the explosion height, the extension time of the corresponding jet in the air, is $t_{H}$, the armor-breaking depth is *L*, and the corresponding armor-breaking time is $t_{L}$; *H* − *b* is a constant.

Through differential Equation (9) with respect to t and with $\frac{dL}{dt_{L}}=u$, Equation (10) can be obtained:

(10) $$t_{H}+t_{L}-t_{b}=\left(t_{H}-t_{b}\right)exp\left(-\int_{v_{j0}}^{v_{j}}\frac{dv_{j}}{v_{j}-u}\right),\;$$

where *H* is the explosion height, the extension time of the corresponding jet in the air is $t_{H}$, the armor-breaking depth is *L*, the corresponding armor-breaking time is $t_{L},\;\mathrm{and}\;v_{j}$ is the velocity of the jet’s microelement about to break the armor, where $v_{j0}=\left(H-b\right)/\left(t_{H}-t_{b}\right)$; *u* is the armor-breaking speed.

We combined $t_{H}+t_{L}-t_{b}=\left(t_{H}-t_{b}\right)exp\left(-\int_{v_{j0}}^{v_{j}}\frac{dv_{j}}{v_{j}-u}\right)$ and $\left(t_{H}+t_{L}-t_{b}\right)=L+H-b$ to obtain Equation (11):

(11) $$L=\left(t_{H}-t_{b}\right)v_{j}exp\left(-\int_{v_{j0}}^{v_{j}}\frac{dv_{j}}{v_{j}-u}\right)-H+b,\;$$

where $u=\frac{v_{j}}{1+\sqrt{\frac{\rho_{j}}{\rho_{t}}}},\;exp\left(-\int_{v_{j0}}^{v_{j}}\frac{dv_{j}}{v_{j}-u}\right)={\left(v_{j}/v_{j0}\right)}^{-1-\sqrt{\rho_{j}/\rho_{t}}}$. Through Equation (9), we have *t* $v_{j0}=\left(H-b\right)/\left(t_{H}-t_{b}\right)$, where $v_{j0}$ corresponds to the case of *L* = 0 and *t* = 0. By introducing this into Equation (11), we can further obtain the armor-breaking depth formula (Equation (12)). That is, the armor-breaking formula of a quasi-steady ideal incompressible fluid.

(12) $$L=\left(H-b\right)\left[{\left(\frac{t_{H}+t_{L}-t_{b}}{t_{H}-t_{b}}\right)}^{\frac{1}{1+\sqrt{\frac{\rho_{j}}{\rho_{t}}}}-1}\right],\;$$

where *H* is the explosion height, the extension time of the corresponding jet in the air is $t_{H}$, the armor-breaking depth is *L*, the corresponding armor-breaking time is $t_{L}$, *H − b* is a constant, $v_{j}$ is the velocity of the jet’s microelement about to break the armor, $v_{j0}$ corresponds to *L* = 0 and *t* = 0, $\rho_{j}$ is the jet’s density, and $\rho_{t}$ is the target’s density.

Considering the strength of the target plate, when the jet’s kinetic energy is lower than the strength of the target plate, the jet cannot break armor, so there is a critical jet velocity $v_{jc}$. The relationship between the armor-breaking velocity and the jet’s velocity is obtained by a Bernoulli equation, as shown in Equation (14).

(13) $$u=\frac{1}{1-\frac{\rho_{t}}{\rho_{j}}}\left[v_{j}-\sqrt{\frac{\rho_{t}}{\rho_{j}}v_{j}^{2}+\left(1-\frac{\rho_{t}}{\rho_{j}}\right)v_{jc}^{2}}\right]\;,\;$$

where $v_{j}$ represents the jet’s velocity, $\rho_{j}$ represents the jet’s density, and $\rho_{t}$ indicates the target plate’s density. Equation (13) can also obtain the armor-breaking depth, as shown in Equation (14). That is, the armor-breaking depth of a quasi-steady incompressible fluid considering the strength:

(14) $$L=\left(t_{0}-t_{A}\right)v_{j}\frac{T_{0}}{T}{\left[\frac{T+\sqrt{T^{2}-{\left(1-c\right)}^{2}v_{jc}^{2}}}{T_{0}+\sqrt{T_{0}^{2}-{\left(1-c\right)}^{2}v_{jc}^{2}}}\right]}^{\frac{-1}{\sqrt{c}}}-H+b,\;$$

where $c=\frac{\rho_{t}}{\rho_{j}},\;T=-cv_{j}+\sqrt{cv_{jc}^{2}+\left(1-c\right)v_{jc}^{2}}$ and $T_{0}=-cv_{j0}+\sqrt{cv_{j0}^{2}+\left(1-c\right)v_{jc}^{2}}$. In order to facilitate the expression and calculation of the formula, T and $T_{0}$ are introduced.

As mentioned earlier in the article, when the jet extends to a certain extent in the air, there will be necking, fracturing, and other phenomena, resulting in the unstable movement of the jet. It will overturn, gradually deviating from the axis, and the penetration ability will be greatly reduced. When the fractured jet penetrates the target, the jet’s velocity is very low, thus the penetration theory must consider the strength and the jet fracture.

According to the jet’s microelement armor-breaking formula dL=udt and $dt=\frac{dl}{v_{j}}-u$, it can be seen that the jet’s microelement at Point A (with a velocity of $v_{j}$) reaches the maximum penetration depth of the target plate, as shown in Equation (15):

(15) $$\left\{\begin{array}{c} dL=-\frac{u}{v_{j}-u}\frac{1}{K}dv_{j}\\ \frac{u}{v_{j}-u}=\frac{v_{j}\;-\;\sqrt{cv_{j}^{2}\;+\;\left(1\;-\;c\right)v_{jc}^{2}}}{-cv_{j}\;+\;\sqrt{cv_{j}^{2}\;+\;\left(1\;-\;c\right)v_{jc}^{2}}}\\ K=-\frac{dv_{j}}{dl} \end{array}\right.\;,$$

The armor-breaking formula under the hydrodynamic theory of fractured jet penetration can be obtained from Equation (15), as shown in Equation (16):

(16) $$\begin{array}{c} L=\frac{1}{Kc}\left[\sqrt{cv_{j0}^{2}+\left(1-c\right)v_{jc}^{2}}-\sqrt{cv_{j}^{2}+\left(1-c\right)v_{jc}^{2}}\right]-\frac{v_{jc}}{K\sqrt{c}}\{\arctan{\left[\frac{cv_{j0}^{2}\;+\;\left(1\;-\;c\right)v_{jc}^{2}}{cv_{jc}^{2}}\right]}^{\frac{1}{2}}\\ -\;\arctan{\left[\frac{cv_{j}^{2}\;+\;\left(1\;-\;c\right)v_{jc}^{2}}{cv_{jc}^{2}}\right]}^{\frac{1}{2}}\;+\;\arctan\left(\frac{\sqrt{c}v_{j0}}{v_{jc}}\right)\;-\;\arctan\left(\frac{\sqrt{c}v_{j}}{v_{jc}}\right)\}\;, \end{array}$$

That is, the formula of quasi-steady incompressible fluid penetration considering the strength of the fractured jet. After the jet breaks, the distance between each section of the jet increases gradually due to the action of air resistance and the difference in velocity. The jet section will turn over due to the action of fracture disturbance and aerodynamic torque, so the penetration ability will be greatly reduced after the jet breaks.Therefore, in the study of actual jet behavior, the penetration formula considering the strength of target plate and the strength of broken jet is more in line with actual jet penetration. Therefore, the calculation result of Equation (16) is more consistent with the penetration depth of the actual warhead.

## 7. Conclusions

In order to study the penetration ability and jet behavior of the post-shock shaped charge jet warhead, the penetration performance of the post-shock shaped charge jet warhead into a target plate, with and without a simulated cabin, was studied. Through numerical simulation and explosion tests, the penetration characteristics were studied and analyzed in detail, and the penetration results of the post-shock shaped charge jet warhead in two cases were obtained. The following conclusions were drawn:

(1) When the shaped energy jet warhead is located behind the control cabin, according to numerical simulation and experimental research, it can penetrate the control cabin and penetrate a 20 mm-thick Q235 steel plate at twice the blast height, indicating that the shaped energy jet has the ability to penetrate light armor after penetrating the control cabin.
(2) If there is no control cabin, when the shaped charge jet warhead directly penetrates a Q235 steel plate at a blast height of 6× the diameter of the shaped charge cover, the maximum penetration depth is 80 mm, indicating that the shaped charge jet warhead has the ability to penetrate thick armor.
(3) Through a combination of experiments and numerical simulation of a shaped charge jet in this work, the penetration process behavior of a shaped charge jet was analyzed theoretically. Considering the target strength and fracturing of the shaped charge jet, the penetration depth was more in line with the actual penetration process of shaped charge jets.
(4) Through the combination of experiments and theory, the formation process of a shaped charge jet was analyzed and disassembled in detail, which provided detailed theoretical and experimental support for improving the performance of armor-breaking warheads in the future.

## Figures and Tables

**Table 1:** Parameters of the liner material.

Materials	ρ (g/cm^3^)	*A* (MPa)	*B* (MPa)	n	c	*m*	T_m_ (K)	T_room_ (K)	c_0_ (cm/μs)	S
Copper	8.93	90	210	0.31	0.025	1.09	1356	293	0.39	1.49

**Table 2:** Material parameters of different target plates.

Material	ρ (g/cm^3^)	*E*(GPa)	NUXY	Yield Stress (MPa)
Q235 steel	7.8	50.9	0.3	355
Aluminum	2.7	71	0.33	90
Nylon 66 plastic plate	1.04	1.07 × 10^−2^		

**Table 3:** Material parameters for the Comp-B explosive.

Materials	ρ (g/cm^3^)	*D* (cm/μs)	*P_cj_* (GPa)	*E* (GPa)	*A* (GPa)	*B* (GPa)	*R*_1_	*R*_2_	ω	$V_{0}$
Explosive	1.717	0.798	29	8.5	524.2	7.678	4.2	1.10	0.34	1

## Data Availability

The data that support the findings of this study are available from the corresponding author upon reasonable request.
